# Two Fatal Obstetrical Cases

**Published:** 1875-02

**Authors:** John Hockenhull

**Affiliations:** Cumming, Forsyth County, Ga.


### TWO FATAL OBSTETRICAL CASES.

BY JOHN HOCKENHULL, M.D., CUMMING, FORSYTH COUNTY, GA.

In compliance with your request to country practitioners to communicate their experience and send cases for publication, I send you an account of the only fatal obstetrical cases that have occurred in my practice since I commenced in the year 1853.

### *CASE I.—PLACENTA PREVIA.*

On the third of February, 1873, 12 M., I was called to consult with Dr. H. P. Riden in the case of Mrs. P., a pluripare, who was in labor. The Doctor informed me that she had been quite feeble during her pregnancy, and so much so during the last five weeks as to be mostly confined to her bed, and to render him extremely anxious in regard to her ability to deliver herself by her natural powers. She had some hemorrhage four days previous to the commencement of labor, and on that morning a copious flow. I found her pale and anemic, pulse feeble and frequent, having an occasional labor pain, accompanied with some nausea.

Shortly after my arrival, a very profuse hemorrhage came on, when I examined the patient, and found the os dilated to the size of a Spanish dollar, and almost as hard as a bone, and the placenta presenting almost centrally. I introduced my hand and separated the placenta from half the circle of the os, and the bag of waters coming down, I ruptured them. The patient by this time was almost exsanguine, bathed in perspiration, suffering great jactitation, retching to vomit, and seemed almost moribund. Notwithstanding the means above mentioned were used to control the hemorrhage, and the head of the child came down on the os and detached placenta, blood continued to flow at each effort to vomit. A strip of cloth, saturated with tinct. chlo. ferri, was at once carried to the os, and strips of cloth were then packed in until the vagina was full. The hemorrhage then ceased, and the patient rallied somewhat under the use of stimulants, nourishment and tinct. opii. At 9 P.M., patient having slept, and being restored as much as we expected her to be—her pains, accompanied with nausea and feelings of fainting, again coming on—I removed the tampon, and on examining found the os dilated, when we determined to deliver her with the forceps. We preferred the forceps, thinking that the shock of turning might extinguish the remaining spark of vitality. At my first examination, neither delivery by the forceps nor version could have been accomplished, on account of the rigidity of the partially dilated os. The head being at the superior strait, occiput towards the right acetabulum, and one-half of the placenta in advance, it was with great difficulty I applied the forceps; but by using the hand and arm as a guide, I succeeded in introducing and locking the forceps, a small dribbling of blood flowing the while. By the time the forceps were applied, which was done as expeditiously as possible, our patient seemed almost in a dying condition, and though further efforts seemed vain, I made three moderate tractive efforts, and readily brought the occiput under the arch of the pubis, when, in view of the child being dead, and being satisfied that our patient was in *articulo mortis*, we desisted from further efforts, and she died in a few moments undelivered.

The feeble health of this lady, and the profuse hemorrhage at the commencement of labor, one would have expected to find the os soft and dilatable; but such was not the case. It was as hard as bone almost. Neither did we find, on separating the placenta so hat there was no further detachment by the dilatation of the os and the rupture of the membranes, thus bringing the head of the child to press on the os and detached placenta, the hemorrhage to cease, as we from our reading were led to expect.

We could easily have delivered our patient after the forceps were applied, but not before death took place; and in view of all the facts of the case, I do not know, even now, that we could have done more to save the life of our patient.

CASE II.—I was called to see Mrs. H., a pluripare, on the night of the ninth of January, 1875, and found her in labor and almost bloodless. She had been having more or less hemorrhage for two weeks, which became profuse about four hours before my arrival, reducing her to a condition of great prostration, accompanied with laborious breathing, coldness of the extremities, great restlessness and a flickering pulse. On examining her per vagina, I found the os undilated and the blood flowing from the vagina, and as no time was to be lost, I introduced a Madam Bovine? speculum, and through it plugged the vagina with strips of soft cloth, in the first of which I wrapped up some tannic acid. This stopped the hemorrhage almost completely, and under the use of stimulants, opiates and nourishment, she rallied. As she was now having no pains or hemorrhage, and was resting well, I desisted from further interference until I could send for another physician. I sent for Dr. P. L. Hughes, who arrived about three o'clock on the following evening. By this time she seemed stronger, and on consulting together, we determined to withdraw the tampon, introduce the hand and make a thorough examination, and, if the os admitted, notwithstanding the only pain she complained of was an aching and feeling of distention at the lower part of the abdomen, to try and deliver her. On introducing the hand, I found it a case of placenta previa—the os fully dilated, part of the placenta protruded and the left shoulder presenting, the scapula to the abdomen of the mother; the head and greater part of the body of the fœtus seemed inclosed by the contracted uterus, which, by its tonic spasm, prevented the hand from reaching them. In front of the axilla, a hand and foot were found; the foot was brought down and the other one searched for, but on account of the contraction of the uterus above mentioned could not be reached. Just here I desire to remark that it is much easier, according to my experience, to

6

read of finding and bringing down the feet than to accomplish it. The mother having no propulsive pains, and being so exhausted as to promise little or no assistance in her delivery, by traction by the foot and giving intervals of rest, Dr. Hughes assisting the process of turning through the abdominal walls, the spasm gave way, and we had the pleasure of delivering her of a dead child, which must have been dead some time before my arrival, as putrefaction had commenced. The mother bore the operation as well as could be expected, considering her condition; there was little or no hemorrhage, and notwithstanding she was almost exhausted when it was completed, she rallied in a few hours—pulse regular, respiration good, slept part of the night, and was doing well when I left her the next morning.

The case continued to do well until about eleven o'clock A.M. January 11th, when, contrary to directions, she was taken up and put upon the night-glass, after which her breathing became laborious; she was very restless; at times suffered with nausea and faitness, which symptoms continued to grow worse, until between eleven and twelve o'clock P.M. she expired.

I did not see her after leaving her in the morning, nor was there any *post mortem* examination, but I opine that there was either further hemorrhage excited by moving her, or an embolism consequent upon the previous extreme exhaustion had thereby been detached, and during the disturbance of the circulation having floated to and become entangled in the pulmonary valves of the heart. I can only attribute the above symptoms and her death to one or the other of the above causes, as I saw no good reason when I left her in the morning why she should not recover. I regard Mrs. H.'s case as being very uncommon, on account of placenta previa, shoulder presentation and tonic spasm of the uterus all occurring in the same case—a terrible ordeal, through which but few parturients ever pass. I know it is uncommon to report our fatal cases, but I have done so hoping that, should some young members of the profession be called on to take charge of life in such peril, my cases may warn them of their responsibility, and cause them to store their minds with such knowledge as will give them decision, promptitude and skill in such emergencies.

